# The Bantam microRNA Is Associated with *Drosophila* Fragile X Mental Retardation Protein and Regulates the Fate of Germline Stem Cells

**DOI:** 10.1371/journal.pgen.1000444

**Published:** 2009-04-03

**Authors:** Yingyue Yang, Shunliang Xu, Laixin Xia, Jun Wang, Shengmei Wen, Peng Jin, Dahua Chen

**Affiliations:** 1 State Key Laboratory of Reproductive Biology, Institute of Zoology, Chinese Academy of Sciences, Beijing, People's Republic of China; 2 Department of Human Genetics, Emory University School of Medicine, Atlanta, Georgia, United States of America; 3 Department of Neurology, Second Hospital of Shandong University, Jinan, Shandong Province, People's Republic of China; 4 Department of Neurology, Qilu Hospital of Shandong University, Jinan, Shandong Province, People's Republic of China; University of Minnesota, United States of America

## Abstract

Fragile X syndrome, a common form of inherited mental retardation, is caused by the loss of fragile X mental retardation protein (FMRP). We have previously demonstrated that *dFmr1*, the *Drosophila* ortholog of the fragile X mental retardation 1 gene, plays a role in the proper maintenance of germline stem cells in *Drosophila* ovary; however, the molecular mechanism behind this remains elusive. In this study, we used an immunoprecipitation assay to reveal that specific microRNAs (miRNAs), particularly the bantam miRNA (*bantam*), are physically associated with dFmrp in ovary. We show that, like *dFmr1*, *bantam* is not only required for repressing primordial germ cell differentiation, it also functions as an extrinsic factor for germline stem cell maintenance. Furthermore, we find that *bantam* genetically interacts with *dFmr1* to regulate the fate of germline stem cells. Collectively, our results support the notion that the FMRP-mediated translation pathway functions through specific miRNAs to control stem cell regulation.

## Author Summary

Fragile X syndrome, the most common cause of inherited mental retardation, results from the loss of functional Fragile X mental retardation protein (FMRP). FMRP is an RNA-binding protein and is known to bind to specific mRNAs and regulate their translation both *in vitro* and *in vivo*. Previous studies have suggested that FMRP is one component of the miRNA pathway involved in miRNA-mediated translational control. In the past we found that, as a translational regulator, dFmrp can modulate the proliferation and fate specification of stem cells in *Drosophila*, likely via the miRNA pathway. However, whether dFmrp could use specific miRNAs to regulate the fate of GSCs has remained unclear. Here, we show that dFmrp is associated with specific microRNAs (miRNAs), including the bantam miRNA, in *Drosophila* ovary. Furthermore, we show that *bantam* genetically interacts with *dFmr1* to regulate the fate of GSCs. Our findings imply that FMRP could utilize specific miRNAs to regulate the translation of its mRNA targets.

## Introduction

Stem cells, which can self-renew and produce different cell types, are known to be regulated by both extrinsic signals and intrinsic factors [1]. In *Drosophila* ovary, a very small population of germline stem cells (GSCs) is maintained in a well-defined microenvironment, which provides an attractive system for investigating the regulatory mechanisms that determine the fate of stem cells [2],[3]. Studies from multiple laboratories have identified the genes that are essential for GSC fate determination [4],[5]. Recently, the microRNA (miRNA) pathway was also found to be required for controlling GSC self-renewal, since mutations in *Dicer-1*, *Ago1*, and *loquacious*, which are involved in miRNA production and function in *Drosophila*, lead to rapid loss of GSCs [6],[7],[8],[9]. MiRNAs could regulate gene expression through translational repression and mRNA degradation by binding to the 3′ untranslated region (UTR) of their target mRNAs [10]. However, the specific miRNAs required for the regulation of GSC self-renewal and fate specification are yet to be determined.

Fragile X syndrome, the most common cause of inherited mental retardation, results from the loss of functional FMRP [11]. FMRP is an RNA-binding protein and is known to bind to specific mRNAs and regulate their translation both *in vitro* and *in vivo* [12]. FMRP is largely cytoplasmic, incorporated into large messenger ribonucleoprotein (mRNP) particles [12]. A growing body of work from several groups now suggests that the microRNA pathway is the major molecular mechanism by which FMRP regulates translation. In *Drosophila* and mammals, FMRP, as well as its autosomal homologs in mammals, FXR1P and FXR2P, is found to be a part of the RNA-induced silencing complex (RISC) [13],[14],[15],[16]. However, what role if any FMRP plays in RNA interference is unclear. In the miRNA pathway, FMRP is associated with miRNAs in both *Drosophila* and mammals, and the genetic interaction between *dFmr1* and the miRNA pathway has been demonstrated in *Drosophila* [13],[17]. Therefore, FMRP is one component of the miRNA pathway involved in miRNA-mediated translational control. Recently, we also showed that *dFmr1* is required for both GSC maintenance and repressing differentiation [17]. Furthermore, we demonstrated that in *Drosophila* ovary, *dFmr1* protein (dFmrp) interacts with Argonaute protein 1 (AGO1), a key component of the miRNA pathway. Hence *dFmr1* could modulate the fate of GSCs, likely via the miRNA pathway. Nevertheless, whether dFmrp could use specific miRNAs to regulate the fate of GSCs has remained unclear.

Here we show that dFmrp is associated with specific miRNAs, including the bantam miRNA, in *Drosophila* ovary. Like *dFmr1*, the bantam miRNA is not only required for repressing primordial germ cells (PGCs), but also functions as an extrinsic factor for GSC maintenance. Furthermore, we show that *bantam* genetically interacts with *dFmr1* to regulate the fate of GSCs. These results support the notion that FMRP-mediated translational control functions through specific miRNA(s) to control stem cell behavior.

## Results

### Identification of Specific miRNAs Associated with *dFmrp* in *Drosophila* Ovary

**Figure 1:**
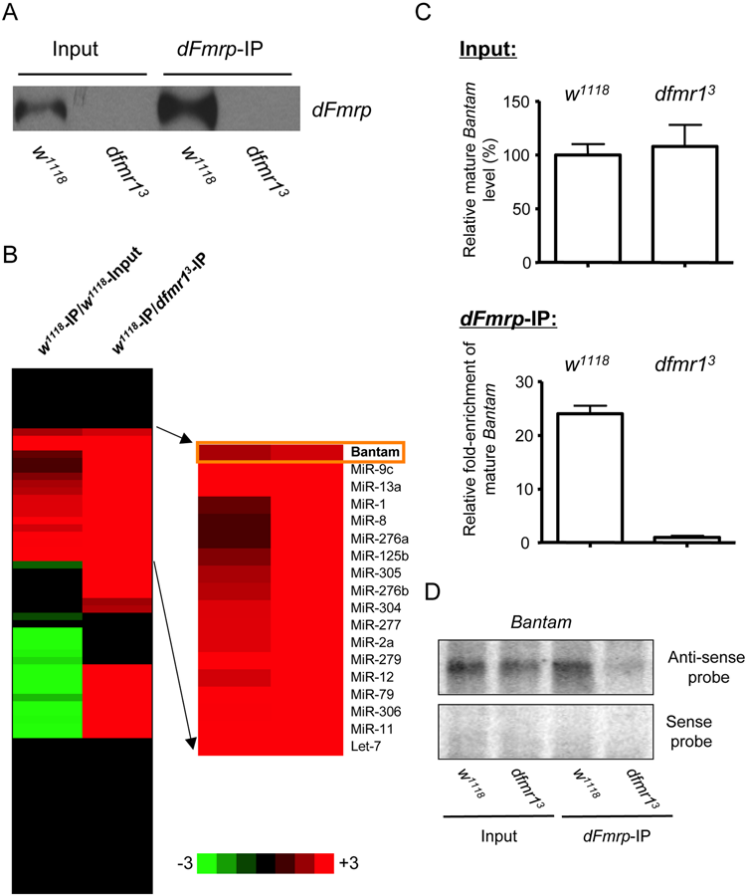
Specific miRNAs associated with *dFmr1* protein in *Drosophila* ovary. (A) Western blot shows that *dFmr1* protein (dFmrp) was immunoprecipitated from wild-type *Drosophila* ovary. A *dFmr1* null mutant (*dfmr1^3^*) was used as a negative control. (B) miRNA TaqMan assays of 72 known *Drosophila* miRNAs were performed in triplicate using both input and IP RNAs from both wild-type and *dfmr1^3^* mutants. The miRNAs that were enriched are shown in progressively brighter shades of red, and the miRNAs that were reduced in IP are shown in progressively brighter shades of green. The miRNAs shown in black were not changed. The fold of the change is indicated on both sides of the scale bar. The miRNAs that are specifically enriched in IP from wild-type ovary are shown. The data represent the average of two biological replicates (two independent immunoprecipitation experiments). (C) TaqMan assays of the bantam miRNA were performed in triplicate, and the enrichment of the bantam miRNA in dFmrp-IP (independent IP experiments from those presented in panel B) from wild-type ovary is shown. (D) Northern blot shows that the bantam miRNA is associated with dFmrp. Northern blots detecting the sense and anti-sense strands of the bantam miRNA in both input and IP RNAs from WT and *dfmr1^3^* mutants are shown.

Given that *dFmr1* plays an important role in the fate determination of germ cells, and that dFmrp physically associates with *dAGO1*, a key component in the miRNA pathway, we proposed that *dFmr1* regulates the fate of GSCs through the miRNA pathway in *Drosophila* ovary [17]. To test this, we first determined whether dFmrp is associated with the endogenous miRNAs. We used the previously developed anti-dFmrp antibody to perform immunoprecipitation from the lysates of both wild-type (WT, *w^1118^*) and *dfmr1^3^* mutant ovaries (Figure 1A) [18]. RNAs from both the immunoprecipitated (IP) complex and input were isolated for miRNA TaqMan assays. We used TaqMan assays available from ABI that could detect a total of 72 known individual miRNAs. To identify the miRNAs specifically associated with dFmrp, we determined the level of each miRNA in both IP and input RNAs from WT and *dfmr1^3^* mutants. We identified the miRNAs that were consistently enriched by more than two-fold in WT-IP over both WT-Input and *dfmr1^3^*-IP in two independent experiments, since such miRNAs are likely to be the ones specifically associated with dFmrp based on previous IP experiments (Figure 1B) [19]. Among the 72 miRNAs examined here, only selective miRNAs were enriched in WT-IP compared with both WT-Input and *dfmr1^3^*-IP, suggesting that dFmrp is indeed associated with specific miRNAs, but not with endogenous miRNAs in general (Figure 1B). Since the bantam miRNA is among those miRNAs specifically associated with dFmrp in ovary, we further confirmed their specific association by quantitative RT-PCR and Northern blot (Figure 1C and 1D). The rest of this study focuses on the role of the bantam miRNA and its potential interaction with *dFmr1* in regulating GSCs.

### Generation of New Alleles for *bantam*

**Figure 2:**
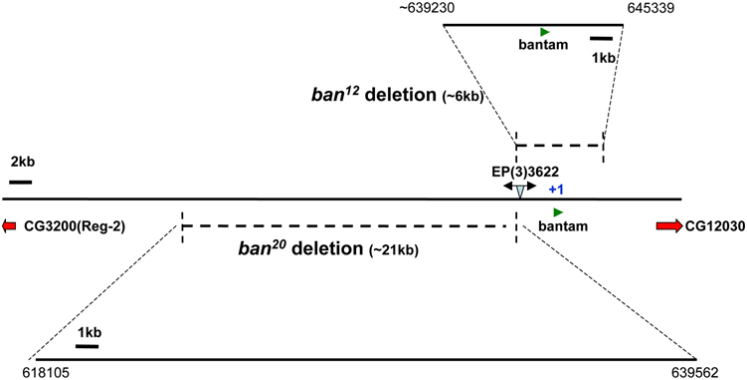
Generation and characterization of new *bantam* mutant alleles. Imprecise mobilization of the P-element from *ban^EP3622^* was carried out to generate stronger alleles for the *ban* gene. One sterile line, *ban^20^*, and one lethal line, *ban^12^*, were isolated. The breakpoints of two mutant lines are shown, with the adjacent genes indicated. Mature bantam miRNA is indicated in green. The chromosomal position and sequence region is based on *Drosophila melanogaster* (R5.13).

To determine whether *dFmr1* regulates germline development through the bantam miRNA, we first explored whether the bantam miRNA plays similar roles to *dFmr1* in repressing primordial germ cells (PGCs) and GSC differentiation during the larval and adult stages. Since the hypomorphic allele of *bantam (ban)*, *ban^EP3622^*, was fertile and exhibited no apparent defects in germ cells, including PGCs and GSCs, we performed a mutagenesis through imprecise mobilization of the P-element from *ban^EP3622^* to generate stronger alleles for the *ban* gene. From 50 imprecise excision lines, we isolated one sterile line, *ban^20^*, and one lethal line, *ban^12^*. Both of these alleles failed to complement the deficiency line of *Df(3L)emc-E12*, which deletes the chromosomal segment that covers the *ban* locus. The breakpoints of these two mutant lines were determined by PCR and DNA sequencing (Figure 2). Furthermore, we detected no mature bantam miRNA by miRNA TaqMan assay in these mutant lines (data not shown). Both *ban^12^* and *ban^12^*/*Df(3L)emc-E12* display lethality at early pupa stage while *ban^20^* and ban^20^/*Df(3L)emc-E12* are viable but sterile. Given that the sterility of flies carrying *ban^20^/Df(3L)emc-E12* and the lethality of flies carrying *ban^12^/Df(3L)emc-E12* could be rescued by transgenic flies carrying P{*banP-ban*} (>90% could be rescued), we conclude that the phenotypes associated with *ban^20^* and *ban^12^* alleles are due to loss of the bantam miRNA.

### The Bantam miRNA Is Required for GSC Maintenance

**Figure 3:**
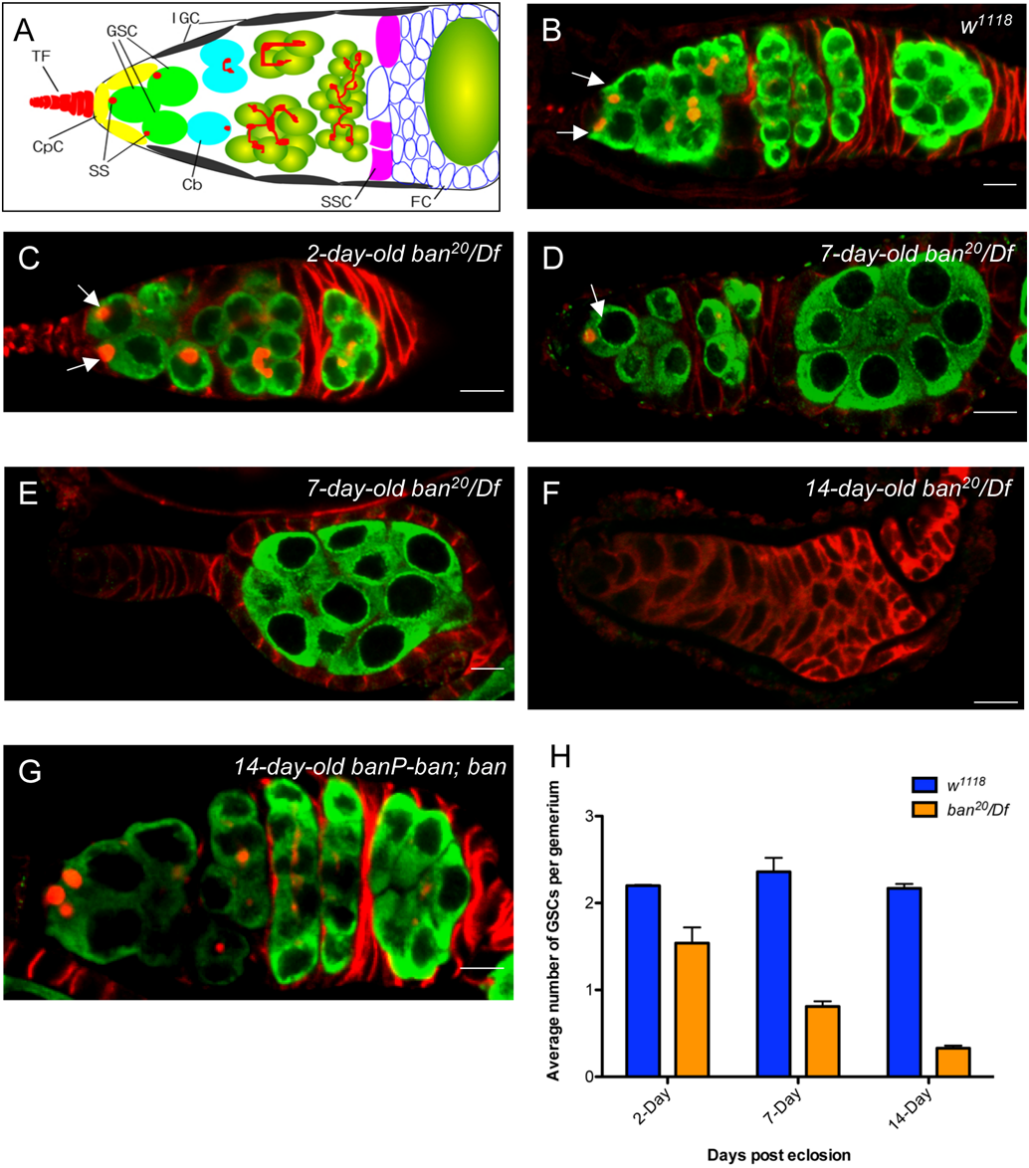
The bantam miRNA is required for GSC maintenance. A. A schematic diagram of a *Drosophila* germarium with different cell types labeled by different colors: GSCs, cystoblast (CB) and cysts, spectrosomes (SS), terminal filaments (TF), cap cells (CPC), inner germarium sheath cells (IGC), follicle cells (FC), SSC (somatic stem cells) and fusomes. B–G: Ovaries from wild-type (B), *ban* mutant flies at different ages (C–F), and *ban* mutants carrying transgene P*{banP-ban}* (G) were stained with anti-*Vasa* (Green) and anti-*Hts* (Red) antibodies. H. Quantitative analyses of the number of GSCs in *ban* mutant; the x-axis shows the day of examination post-eclosion, while the y-axis shows the average number of GSCs per germarium in *ban* mutants and WT. *P<0.001* when *ban* mutant was compared with WT at different time points. Arrows indicate GSCs.

A typical *Drosophila* ovary is composed of 16–20 distinct units known as ovarioles. Each ovariole consists of an anterior functional unit, called a germarium, and a linear string of differentiated egg chambers posterior to the germarium. At the tip of the germarium, GSCs normally divide asymmetrically to ensure that one daughter cell remains attached to the niche cells for self-renewal, while the other is displaced from the niche, becoming a cystoblast (CB) that initiates differentiation and sustains oogenesis (Figure 3A) [20]. We have previously shown that *dFmr1* is involved in the regulation of GSC fate [17]. To compare the biological effects of *ban* with *dFmr1* in GSCs, we examined whether *ban* is also required for GSC maintenance by quantifying the number of GSCs in *ban* mutant germaria at different ages, as we described previously, by staining them with anti-*Vasa* and anti-*Hts* antibodies. *Vasa* staining can specifically visualize all germ cells during oogenesis, while *Hts* is preferentially rich in fusome, a germ cell-specific organelle that is morphologically spherical in primordial germ cells and GSCs/cystoblasts, but branched in differentiated cysts. GSCs can be reliably recognized at the tip of the germarium by their position of direct contact with cap cells or base cells of the terminal filament and the anterior localization of spherical fusomes (also called spectrosomes) [4]. Using anti-*Vasa* and anti-*Hts* antibodies, we stained the WT and *ban* mutant ovaries to visualize germ cells and fusomes, respectively. As shown in Figure 3, in wild-type ovaries, there were an average of ∼2–3 GSCs per germarium (Figure 3B and 3H). However, the two-day-old *ban^20^/Df(3L)emc-E12* germaria contained an average of 1.54 GSCs (n = 147, with about 60% of germaria having two GSCs (Figure 3C), and the other containing one or no GSCs), indicating that *ban* is required for GSC establishment or GSC maintenance. Furthermore, the seven-day-old and 14-day-old *ban* mutant germaria had an average of 0.80 (n = 95) and 0.33 (n = 141) GSCs (Figure 3D, E, F, and H), respectively. This phenotype could be rescued by the expression of the bantam miRNA (Figure 3G). These data together suggest that the loss of *ban* resulted in the progressive loss of GSCs. Thus, these results demonstrate that the bantam miRNA is required for maintaining GSCs in *Drosophila* ovary.

### The Bantam miRNA Is Required for Repressing PGC Differentiation

**Figure 4:**
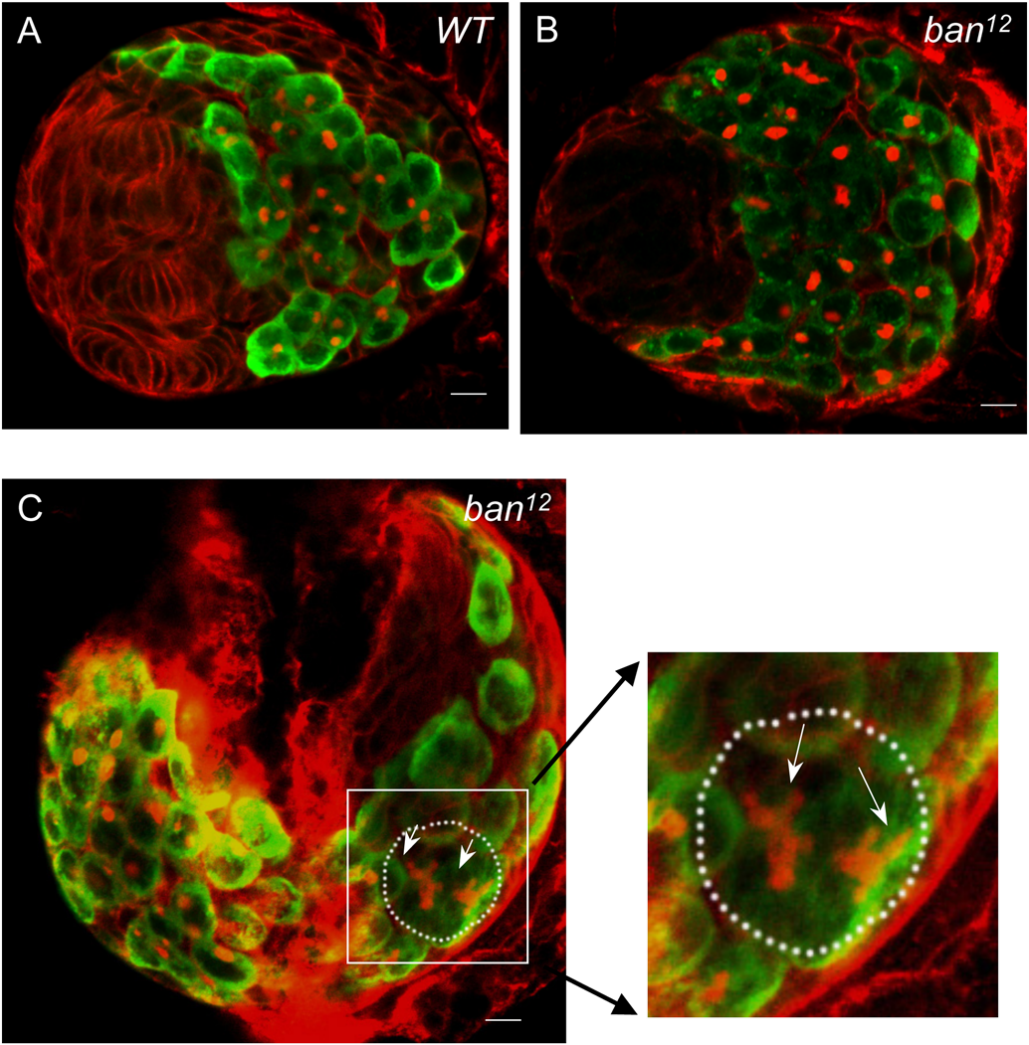
The bantam miRNA is required for repressing primordial germ cell (PGC) differentiation. Gonads from the third-instar larvae of *w^1118^* (A) and *ban^12^* mutants (B, C, and C inset) were stained with anti-*Vasa* and anti-*Hts* antibodies. Anti-*Hts* (Red) was used to outline gonads and morphology of fusomes, while anti-*Vasa* (green) was used to visualize all germ cells. PGCs carrying a single round fusome are indicated by arrows, while differentiated germ cells are indicated by arrowheads. Scale bar represents 10 µm.

Our previous study demonstrated that *dFmr1* plays a role in repressing PGC differentiation [17]. To determine whether *ban* plays a similar role in PGC development, we examined the behavior of PGCs in *ban* mutants. PGCs are the precursors of germ cells during the three larval stages; the number of PGCs simply grows from 12 cells to more than 100 cells, but without further differentiation before the pupa stage. Using anti-*Vasa* and anti-*Hts* antibodies as previously described, we stained the third instar gonads of both WT and *ban* mutants to visualize germ cells and fusomes, respectively [21]. As shown in Figure 4A, in WT gonads from late third-instar larvae, ∼80% PGCs carried a single spherical fusome, and the other PGCs were dividing with two spherical fusomes associated between two PGC cells. By contrast, in the *ban^12^* mutant, most gonads (84%, n = 25) from the third-instar larvae contained differentiated PGC clusters that were marked by branched fusomes (Figure 4B and 4C), reminiscent of other allelic combinations of *ban* (*ban^12^/Df(3L)emc-E12*) (75%, n = 12). Together, these results suggest that, like *dFmr1*, *ban* plays a similar role in repressing PGC differentiation.

### The Bantam miRNA Plays a Non-Autonomous Role in GSC Maintenance

**Figure 5:**
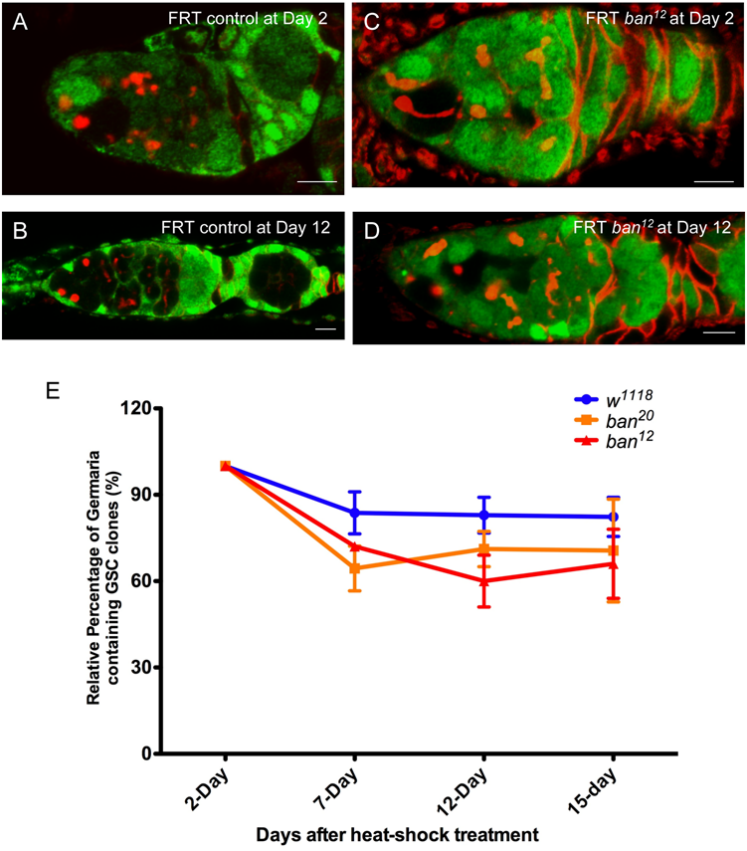
The bantam miRNA plays a non-autonomous role in GSC maintenance. GSC clones were induced by heat-shock treatment in adult female flies. Ovaries from FRT control flies (A and B) and FRT, *ban* flies (C and D) were dissected at day 2 and day 12 following heat-shock treatment; GSC clones were identified by the lack of GFP expression. Scale bar represents 10 µm. (E) Relative percentages of negatively GFP-marked GSC clones in FRT control and two FRT; *ban* null alleles at days 2, 7, 12, and 15 AHST are shown. *P>0.5*.

The loss of germline stem cells in *ban* mutant ovaries indicates that *ban* is required by either GSCs or somatic cells. To analyze whether *ban* functions as a cell-autonomous factor in maintaining GSC fate, we used a FLP-FRT–mediated mitotic recombination technique to generate marked mutant GSCs [17],[22], then calculated the life span of the marked mutant GSCs by quantifying their loss rate. The marked mutant GSCs were identified by a lack of GFP fluorescence in the nuclei and by their positions directly attaching to the base cells of the terminal filament or cap cells. The *ban* loss-of-function alleles (*ban^20^ and ban^12^*) were used to generate marked mutant GSC clones for an analysis of *ban* function in GSCs. The rates of GFP-marked GSCs were measured at two days, seven days, 12 days, and 15 days after heat-shock treatment (AHST). As shown in Figure 5E, compared with FRT control GSC clones, the marked clone rates of both *ban^20^* and *ban^12^* were not reduced significantly during the testing period, indicating that *ban* is dispensable for GSCs. Together with the phenotypic analyses of *ban*, our data indicate that, as with *dFmr1*, *ban* functions as an extrinsic factor for GSC maintenance (Figure 5).

### *Bantam* Genetically Interacts with *dFmr1*

**Figure 6:**
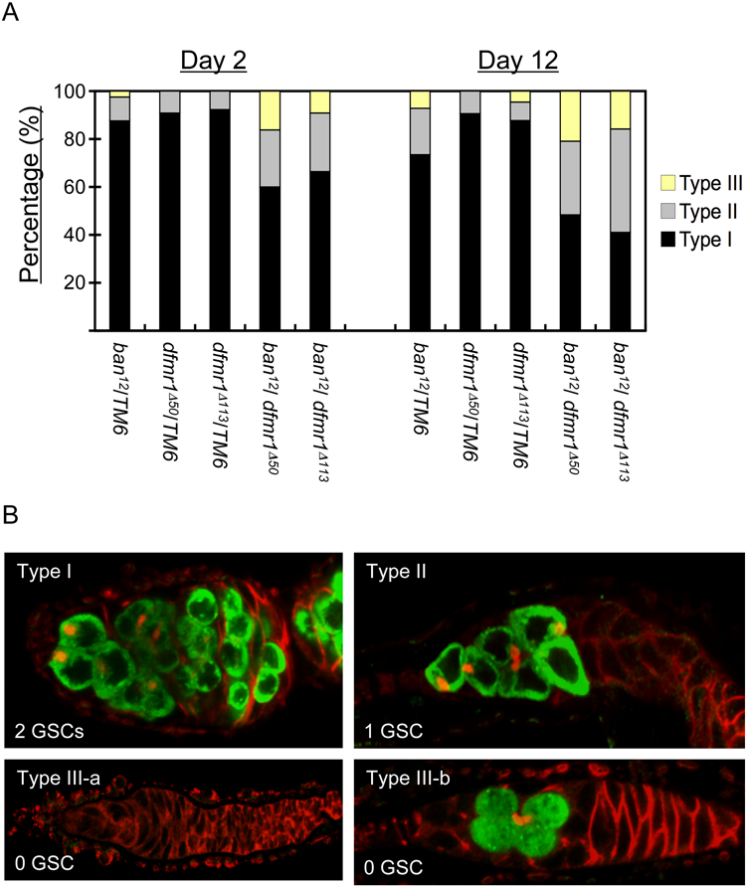
*Bantam* genetically interacts with *dFmr1* in modulating GSCs. (A) The number of GSCs per germarium was measured from *dfmr1* heterozygotes, *ban* heterozygotes, and *dfmr1*, *ban* double heterozygotes at day 2 and day 12 post-eclosion, respectively. *P<0.01* when the percentage of type II and III germaria of the trans-heterozygotes were compared with those of either WT or single heterozygotes. (B) Ovaries from the genotypes above were stained with anti-*Vasa* (Green) and anti-*Hts* (Red); germaria carrying two GSCs, one GSC, and no GSCs were referred to as type I, II, III-a and III-b, respectively.

Given that *ban* plays a similar role to *dFmr1* in the regulation of GSC fate and that *Ago1* genetically enhances the phenotype of *dFmr1* in the maintenance of GSCs [17], we next investigated whether *ban* genetically interacts with *dFmr1*. Interestingly, we found that compared with the control flies, double heterozygotes of *dfmr1* and *ban* displayed significantly reduced fertility in female flies (Table 1), which suggests that, besides the physical association, *ban* also genetically interacts with *dFmr1* in *Drosophila* oogenesis. To test whether the partial loss of both the ban miRNA and *dFmr1* could affect the normal maintenance of GSCs, we further quantified the number of GSCs in the single or double heterozygotes of *ban* and *dfmr1* mutants at days two and 12 after eclosion. As shown in Figure 6, the double heterozygotes displayed a significant increase in the number of germaria with one or no GSCs, suggesting that a reduction of both *ban* and *dFmr1* leads to greater loss of GSCs. In both experiments, we found that the balancer chromosomes (TM6) did not cause any phenotype while they are in trans to either *ban* or *dfmr1* null allele (data not shown). These data suggest that *dFmr1* could potentially regulate the fate of GSCs through the bantam miRNA.

**Table 1:** Relative fertility of different mutant flies.

Genotype	Percentage of fertility
*w^1118^*	98.9% (87/88)
*ban^12^/TM6*	97.5% (78/80)
*dfmr1^Δ50^/TM6*	98% (49/50)
*dfmr1^Δ113^/TM6*	97.8% (45/46)
*ban^12^/dfmr1^Δ50^*	7.7% (5/65)*
*ban^12^/dfmr1^Δ113^*	4.6% (3/65)*

Individual female was crossed with wildtype (*w^1118^*) male flies and the number of fertile eggs was quantified.

*: *P<0.01* when the trans-heterozygotes were compared with either WT or single heterozygotes.

## Discussion

Fragile X syndrome, the most common cause of inherited mental retardation, is due to the loss of functional FMRP [11]. As an RNA-binding protein, FMRP is known to regulate the translation of specific mRNAs [23]. Recent work has suggested that FMRP could regulate translation via the miRNA pathway [24],[25]; however, the experimental evidence to elucidate the specific role, if any, FMRP plays in small RNA-mediated post-transcriptional gene regulation is still lacking, and whether specific miRNAs are used in FMRP-mediated translational control remains to be determined. In this study, we found that dFmrp is associated with specific miRNAs, including the bantam miRNA, in *Drosophila* ovary. Interestingly, like *dFmr1*, *ban* also functions as an extrinsic factor in the maintenance of GSCs. Even more importantly, though, we show that *bantam* genetically interacts with *dFmr1* to regulate the fate of GSCs. These data together suggest that dFmrp can use specific miRNA(s) to regulate the behavior of stem cells. These findings also implicate that FMRP could be associated with selective miRNAs, and utilize specific miRNA(s) to regulate the translation of specific mRNA targets.

Studies of *Drosophila* germline stem cells have yielded heuristic examples to help us understand the molecular regulatory mechanism of stem cells. Previous investigations have demonstrated that the self-renewal of GSCs requires both extrinsic and intrinsic mechanisms. Gene products, such as Piwi, Yb, Dpp, and Gbb, are produced from niche cells and function as extrinsic factors for GSC maintenance. On the other hand, intrinsic factors, including Nanos (Nos), Pumilio (Pum), the Dpp-dependent receptors, and transcription factors, are also important for GSC self-renewal [2],[4]. In *Drosophila*, *dFmr1* is known to be required for cyst formation and oocyte specification, potentially via the regulation of *Orb* mRNA translation [26]. Our recent study demonstrated that *dFmr1* is involved in modulating the fate of GSCs [17]. The phenotypic assay of *dfmr1* mutants revealed that the loss of *dFmr1* function leads to a defect in the maintenance of GSCs; however, clonal analyses of *dFmr1* via FLP/FRT-mediated recombination demonstrated that *dFmr1* is dispensable in GSC fate regulation, suggesting *dFmr1* functions as an extrinsic factor. In this paper, we found that the bantam miRNA is associated with dFmrp in *Drosophila* ovary. To determine the role of the bantam miRNA, we generated null alleles of *ban* and examined the role of the bantam miRNA in the maintenance and fate specification of GSCs. Interestingly, we found that *ban* plays a similar role to *dFmr1* in the regulation of GSC fate. More importantly, the double heterozygotes of *ban* and *dFmr1* mutants displayed a greatly reduced number of GSCs per germarium and lower fertility in general, suggesting that *ban* strongly interacts with *dFmr1* genetically in regulating the maintenance and fate specification of GSCs. Furthermore, the transgene *P{banP-ban}* could not rescue the GSC phenotype associated with the loss of *dFmr1* (data not shown). The role of *ban* in GSC maintenance and its genetic interaction with *dFmr1* appear specific since another miRNA that is associated with dFmrp, miR-1, has no effect on GSCs (Chen and Jin, unpublished data). Although it is possible that *ban* could potentially function upstream of *dFmr1*, however, given the strong genetic interaction that we observed, it is more likely that *ban* functions in concert with *dFmr1* to repress GSC differentiation by repressing the translation of common target(s) in the *dFmr1*-dependent pathway.

The role of the miRNA pathway in GSC fate specification has already been explored. Previous work including our own has demonstrated that *Dicer1*, *Loqs*, and *Ago1*, key components of the miRNA pathway, play intrinsic and essential roles in the maintenance of GSCs [6],[7],[8],[9]. In this study, we chose to focus on a specific miRNA, the bantam miRNA. We found that, besides the intrinsic role of the miRNA pathway in general, specific miRNAs, such as the bantam miRNA, could also function as a niche factor to regulate GSCs. Given our findings, the next important step becomes identifying the mRNA target(s) of the bantam miRNA that could regulate GSCs.

In summary, here we show that, as an extrinsic factor regulating GSCs, dFmrp is selectively associated with specific miRNAs, including the bantam miRNA, in *Drosophila* ovary. The strong genetic interaction between *ban* and *dFmr1* in the regulation of GSCs suggests that dFmrp could use the bantam miRNA to regulate the translation of specific mRNAs in ovary, which in turn modulates the behavior of GSCs. Identifying those mRNAs that are co-regulated by dFmrp and the bantam miRNA in *Drosophila* ovary will provide an important system for further study of the role(s) that dFmrp plays in miRNA-mediated translational control.

## Materials and Methods

### *Drosophila* Genetics

All flies were maintained under standard culture conditions. *dfmr1^3^*, *dfmr1^delta113^* and *dfmr1^delta5^* were null *dFmr1* alleles as described previously [18],[27]. *ban^EP3622^* is a P-element insertion line obtained from the Szeged stock center. The deficiency line *Df(3L)emc-E12*, containing the deletion of the chromosomal segment covering the *ban* locus came from Bloomington Stock center. Two *ban* alleles, *ban^12^*and *ban^20^*, were generated by imprecise excision from *ban^EP3622^*. *ban^12^* carried an ∼6-kb deletion including the *ban* core sequence. *ban^20^* is a hypomorphic allele that carried a ∼21-kb deletion (−24.2 kb to −2.7 kb) upstream of the *ban* gene. The transgene P{*banP-ban*} with 7.2-kb genomic rescue fragment, in which about 500 bp *ban* primary transcript was under the control of the 6.7-kb *ban* promoter (3.5-kb upstream and 3.2-kb downstream), was used to rescue the *ban* mutant phenotype. The following primers were used to map the breakpoints of ban^12^ and ban^20^ alleles.

Forward primers:

EP-6K: gtagcttgca gtgggcttac atg

EP-7.5K: cggagtactg tcctccgggc tgg

ban-24.5K: tcattgaccaaatcccaacgcaag

ban-4K: attccagaaattcttgcg

Reverse primers:

EP: ccaccttatgttatttcatcatg

ban-hoop-2K: aggttaggatcgtcgagt

ban-hoop-4.2K: gcgcgatccgaagtcgagactacat

ban-hoop-3K: gtgttgtaatctacggaac

### Immunohistochemistry and Microscopy

Ovaries were prepared for reaction with antibodies as described previously [17]. The anti-*Vasa* antibody (Santa Cruz) was used at a 1∶200 dilution, and monoclonal anti-*Hts* antibody was used at a 1∶500 dilution. Secondary antibodies used were goat anti-mouse Alexa 568, goat anti-rabbit Alexa 488, and goat anti-rat Cy3 (Molecular Probes), all at 1∶200. All samples were examined by Zeiss Microscope, and images were captured using the Zeiss Two Photon Confocal LSM510 META system. Images were further processed with Adobe Photoshop 6.0.

### Phenotypic Assay for Quantification of GSC Maintenance in Mutant Adult Ovaries

Ovaries isolated from *w^1118^* and homozygous mutant flies of different ages were incubated with anti-*Hts* antibody and anti-*Vasa* antibody to identify terminal filament cells, fusomes, and germ cells. We scored as GSCs any *Vasa*-positive germ cells at the anterior position that appeared close to cap cells or to the basal cells of terminal filaments and also carried spherical fusomes at the anterior position or extending fusomes.

### Germline Clonal Analyses

FLP/FRT-mediated recombination was used to generate *dfmr1* mutant GSC and PGC clones. To generate GSC clones, 3-day-old females, *hs-flp*; *FRT79D*, *ubi-gfp/FRT79D*, *ban*, underwent heat-shock treatment at 37°C for 60 min twice daily at 12-h intervals (*hs-flp*; *FRT79D*, *ubi-gfp/FRT79D* as control). After 5–6 days of heat-shock induction monitored by GSC clone efficiency of control, ovaries were dissected for quantification of GSC clones at day 2, day 7, day 12 and day 15 of the post-clonal induction. The % of GSC clones measured at day 2 was also calculated as the initial rate (100 of relative %). GSC clones were identified by a lack of GFP fluorescence in the nucleus and by the carrying of an anterior-positioned dot fusome (spectrosome).

### Immunoprecipitation of dFmrp from Fly Ovary

About 150 adult WT and *dfmr1* mutant fly ovaries were dissected in PBS and homogenized in 1 ml of ice-cold lysis buffer (10 mM Tris, pH 7.4, 150 mM NaCl, 30 mM EDTA, 0.5% Triton X-100) with 2× complete protease inhibitors. All further manipulations of the ovary lysates were performed at 4°C or on ice. Nuclei and debris were pelleted at 3,000×g for 10 min; the supernatant was collected and raised to 300 mM NaCl and clarified at 14,000×g for 30 min. The resulting supernatant was precleared for 1 h with 100 µl recombinant protein G agarose (Invitrogen) (washed with lysis buffer first). An aliquot of precleared input was saved for RNA extraction (200 µl) and protein analysis (100 µl). We incubated 15 µg of Monoclonal Anti-dFMR1 Clone 6A15 (Sigma) with recombinant protein G agarose at 4°C for 2 h and washed 3 times with lysis buffer. RNase Inhibitors (Invitrogen) were added to the remaining lysates. The precleared lysates were immunoprecipitated with antibody-coated recombinant protein G agarose at 4°C overnight. After a third wash with the lysis buffer, 10% of the immunoprecipitate was saved for protein analysis. The remainder was washed one more time, and the immunoprecipitate was resuspended into Trizol (Invitrogen) for RNA isolation or kept at −80°C until further processing. Western blot analysis was performed using anti-dFmrp antibody 5A11 (Developmental Studies Hybridoma Bank at the University of Iowa) and detected with horseradish peroxidase-coupled secondary antibody (Sigma) using the chemiluminescent (GE) method.

### miRNA TaqMan Assay

Total RNA was isolated from the WT and *dfmr1* mutant ovary IP as well as Input using Trizol. TaqMan MicroRNA Assays detecting 72 known individual *Drosophila* miRNAs were obtained from ABI (ABI). cDNA was prepared with a High-Capacity cDNA Archive Kit (ABI): 10× reaction buffer 0.5 µl; dNTP mix (100 mM) 0.05 µl; Recombinant RNase inhibitor 0.07 µl; MMLV reverse transcriptase 0.33 µl; RT TaqMan assays primer (5×) 1 µl; RNA sample 1 µl; and nuclease-free water to 5 µl. This was performed at 16°C for 30 min and at 42°C for 30 min, terminated at 85°C for 5 min and 4°C forever. cDNA was prepared for the endogenous control Dm_RpL32_1_SG by QuantiTect Primer Assay (Qiagen) according to the manufacturer's instructions. Real-time PCR was conducted with 2× SYBR Green Master Mix (ABI) as amplification of RPL32. For Real-time PCR of TaqMan MicroRNA Assays, we used TaqMan MicroRNA Assay Primer (20×) 0.5 µL; cDNA (undiluted) 1.33 µl; TaqMan 2×Universal PCR Master Mix, 5 µl; nuclease-free water 3.17 µl. Real-time PCR reactions performed in triplicate with MicroAmp optical 96-well plates using 7500 Fast Real-Time PCR System (ABI) with the following conditions; an initial step of 10 min at 95°C, followed by 40 cycles of 15 s at 95°C, 1 min at 60°C.

### Northern Blot of miRNAs

RNAs were separated on 15% TBE urea gel, transferred, and UV-cross-linked to nylon membrane (Osmonics, Inc). ^32^P-UTP–labeled probes were prepared with the Ambion mirVana miRNA Probe Construction Kit. Membranes were prehybridized at 65°C for 1 h and hybridized for 12–16 h at room temperature (RT). Membranes were then washed 3 times at RT and 2 times at 42°C. Membranes were exposed and scanned with a Typhoon 9200 PhosphorImager (Amersham Biosciences).

### Statistical Method

We performed post-hoc *t*-tests (two-samples assuming equal variances) to determine significance, and indicated *P* values.
